# Minimal important change of the Western Ontario Osteoarthritis of the Shoulder (WOOS) index in patients with glenohumeral osteoarthritis and rotator cuff tear arthropathy

**DOI:** 10.1007/s00402-025-05778-z

**Published:** 2025-02-15

**Authors:** Josefine Beck Larsen, Theis Muncholm Thillemann, Antti P. Launonen, Helle Kvistgaard Østergaard, Thomas Falstie-Jensen, Bakir Sumrein, Srdjan Zivanovic, Steen Lund Jensen, Inger Mechlenburg, Ville Ponkilainen

**Affiliations:** 1 Department of Clinical Medicine, Aarhus University, Palle Juul-Jensens Boulevard 99, Aarhus N, DK-8200 Denmark; 2 Department of Orthopaedic Surgery, Aarhus University Hospital, Palle Juul-Jensens Boulevard 99, Aarhus N, DK-8200 Denmark; 3 Department of Orthopaedic Surgery, Tampere University Hospital, Teiskontie 35, Tampere, FI-33520 Finland; 4 Department of Orthopaedic Surgery, Viborg Regional Hospital, Heibergs Alle 4 F, Viborg, DK-8800 Denmark; 5 Department of Orthopaedics, Aalborg University Hospital, Hobrovej 18-22, Aalborg, DK-9000 Denmark; 6 Department of Clinical Medicine, Aalborg University, Selma Lagerløfs Vej 249, Gistrup, Aalborg, DK-9260 Denmark; 7 Department of Public Health, Aarhus University, Aarhus, Denmark

**Keywords:** Minimal important change (MIC), Patient reported outcome measures (PROM), Total shoulder arthroplasty, Anatomical total shoulder arthroplasty (TSA), Reverse total shoulder arthroplasty (RSA), The Western Ontario Osteoarthritis of the Shoulder Index (WOOS), Disabilities of the arm shoulder and hand (DASH)

## Abstract

**Introduction:**

The Minimal Important Change (MIC) for patient-reported outcome measures is the value that describes the smallest improvement considered worthwhile by patients. To the best of our knowledge, no MIC of the Western Ontario Osteoarthritis of the Shoulder Index (WOOS) score or the Disabilities of the Arm Shoulder and Hand (DASH) has been reported using the anchor-based predictive modeling approach based on patients with glenohumeral osteoarthritis or rotator cuff tear arthropathy. The aim of this study was to determine the MIC for WOOS and DASH in a cohort of patients with glenohumeral osteoarthritis or rotator cuff tear arthropathy treated with a total shoulder arthroplasty.

**Materials and methods:**

Data on 231 patients were collected at four hospitals in Denmark and Finland. Data were collected at baseline and 12 weeks after surgery. At 12 weeks, the patients were asked about their perceived overall improvement after surgery measured by the Patient Global Impression of Change (PGI-C). The MIC was estimated for the WOOS and DASH using the adjusted predictive modeling approach with the PGI-C as an anchor.

**Results:**

Of the 231 included patients, 104 were included in the MIC analysis. Patients had a mean age of 71 years and 56% were women. The estimated adjusted MIC for the WOOS score was 13.3 (-6.2; 23.3) and 7.2 (12.8; 1.7) for DASH.

**Conclusion:**

For patients with glenohumeral osteoarthritis or rotator cuff tear arthropathy treated with a total shoulder arthroplasty, the estimated MIC for was 13.3 for WOOS and 7.2 for DASH. The estimates show wide confidence intervals, which could be due to the low sample size but could also indicate a large heterogeneity within the patient group.

## Introduction

Patient reported outcome measures are widely used in clinical trials and as a quality-of-care measure in various registries and National Arthroplasty Registries [1–3]. Multiple patient-reported outcome measures (PROMs) have been developed to measure the symptoms and disability among patients. Commonly used PROMs for shoulder patients include the Western Ontario Osteoarthritis of the Shoulder Index (WOOS) and the Disabilities of the Arm Shoulder and Hand (DASH) [1, 4, 5]. The WOOS score is a PROM used in the Nordic shoulder arthroplasty registries [1]. The WOOS score was developed to evaluate the symptoms of patients suffering from glenohumeral osteoarthritis [6]. DASH is an upper-extremity-specific PROM measuring symptoms and physical function developed for patients with upper extremity musculoskeletal conditions [7].

Using PROMs in research requires a meaningful interpretation from the patient’s perspective of treatment effects. The minimal important change (MIC) value offers such an interpretation and is defined as the minimal change score that is considered important by the average patient [2, 8].

The reported MIC values for WOOS (0-100, 100 best) following total shoulder arthroplasty ranges from 8.2 to 12.3 points measured preoperatively to one year after surgery [9, 10]. No previous studies have estimated MIC for DASH for patients undergoing total shoulder arthroplasty. In various other shoulder conditions, the MIC value for DASH has been estimated from 4.4 to 13 points with different follow-up times using an anchor-based approach [11–16]. With different methodological approaches to determine the MIC values [2], there is a need for further studies investigating the MIC values with different anchor-based methods.

The primary aim of this study was to determine the MIC for the WOOS score for patients with glenohumeral osteoarthritis or rotator cuff tear arthropathy, treated with total shoulder arthroplasty. The secondary aim was to determine the MIC for the DASH score in the same patient population.

## Materials and methods

### Design

This study is a prospective observational cohort study of patients with glenohumeral osteoarthritis or rotator cuff tear arthropathy, treated with a total shoulder arthroplasty.

### Study setting

Patients were recruited from Orthopedic Departments at Aarhus University Hospital, Aalborg University Hospital and Viborg Regional Hospital in Denmark and at Tampere University Hospital in Finland. Physiotherapists and orthopedic surgeons at the participating departments assessed eligibility of all patients and eligible patients were asked to complete the questionnaires (paper or electronical) preoperatively and 3 months postoperatively.

### Participants

Patients scheduled to primary total shoulder arthroplasty with glenohumeral osteoarthritis or rotator cuff tear arthropathy were invited to participate from March 2020 to January 2023. Patients were allowed to participate with the first shoulder undergoing surgery.

### Eligibility criteria

Inclusion criteria:

- Patient ≥ 55 years.
- Primary glenohumeral osteoarthritis eligible for anatomical total shoulder arthroplasty or rotator cuff tear arthropathy eligible for reverse shoulder arthroplasty.

Exclusion criteria:

- Previous shoulder fracture.
- Planned other upper extremity surgery within six months.
- Rheumatoid arthritis and other types of inflammatory arthritis.
- Cancer diagnosis and receiving chemo-, immuno- or radiotherapy.
- Neurological diseases (e.g. previous stroke, multiple sclerosis, Parkinson’s, Alzheimer’s).
- Other reasons for exclusion (i.e. mentally unable to participate ect.)
- Inability to understand the written local language (Danish or Finnish).

### Outcome measures

Demographic data was collected at baseline including sex, age, hand dominance and index shoulder, furthermore surgical type (RSA or TSA) and hospital site was registered. In addition, patients completed WOOS and DASH at baseline. At 3 months follow-up patients completed WOOS, DASH and the Patient Global Impression of Change (PGI-C).

### Western ontario osteoarthritis of the shoulder index (WOOS)

The WOOS score is a disease-specific PROM for shoulder-related Quality-of-Life developed for patients with glenohumeral osteoarthritis [6]. The WOOS index consists of 19 items divided into four domains: physical symptoms (6 items), sport and work (5 items), lifestyle (5 items) and emotions (3 items). The items are answered on a visual analogue scale ranging from 0 to 100. The total raw score ranges from 0 to 1900, with 1900 indicating the worst score. The raw scores are often converted into a percentage score where 100% represents a completely healthy shoulder [6]. The WOOS has been translated and cross-culturally validated in Danish, but not in Finnish [17]. In the original WOOS questionnaire, the scoring manual did not facilitate the calculation of a composite score in the presence of missing items. With WOOS being utilized across registries and clinical trials, it is problematic that a few missing data are not allowed. Therefore, we defined that to be included > 50% of items must be answered for each subscale: physical symptoms (3 items), sport and work (3 items), lifestyle (3 items) and emotions (2 items). Allowing for a calculation of the total percentage score following the formula:

$$percentage\,score = {\matrix{\left({{\rm{number}}\,{\rm{of}}\,{\rm{answered}}\,{\rm{items*}}100} \right) \hfill \cr - {\rm{total}}\,{\rm{aggregate}}\,{\rm{score}} \hfill \cr} \over {{\rm{number}}\,{\rm{of}}\,{\rm{answered}}\,{\rm{items}}}}$$.

### Disabilities of the arm shoulder and hand (DASH)

The DASH score is an upper-extremity specific PROM measuring symptoms and physical function for patients with musculoskeletal conditions of the upper-extremities [7]. The DASH consists of 30-items assessing the degree of difficulty performing different physical activities due to arm, shoulder, and hand problems (21 items), symptoms from the arm, shoulder, and hand problems (5 items), difficulties experienced in social life, work, sleep, and self-perception (4 items). The responses are presented as 5-point Likert scales. The items are summarized into a total score from 0 to 100, with 100 indicating severe disability, allowing for three missing items. The questionnaire has been translated and cross-culturally validated in Finnish [18] and Danish [11, 19].

### Patient global impression of change (PGI-C)

The PGI-C is a rating scale where patients are asked a single question at follow-up to evaluate the change of symptoms reported by the patients [20, 21]. The question is: “Since the start of the study, my overall status is:” answered on a 7-point scale ranging from very much improved to very much worse (Table 1). The questionnaire has been translated and cross-culturally validated in Finnish and Danish [22].

**Table 1:** Patient global impression of change (PGI-C) response options and classification into importantly improved or not importantly improved

**Importantly improved**
1. Very much improved
2. Much improved
**Not importantly improved**
3. Minimally improved
4. No Change
5. Minimally worse
6. Much Worse
7. Very much worse

### Statistical analysis

Patient demographics and clinical data are presented as mean and standard deviations (SD) for normally distributed and median and interquartile range (IQR) for non-normally distributed continuous variables, and categorical variables are presented as numbers (proportion). The association between the WOOS and DASH change score and responses on the anchor question was investigated with Pearson correlation. A minimal correlation of 0.3 is considered appropriate in using the anchor question for the MIC determination [2].

MIC values were calculated using the anchor-based predictive modeling as described by Terluin et al. [23]. The WOOS and DASH change scores were anchored to the PGI-C response at 3-month follow-up. Due to its methodological advantages, the predictive modelling method (MIC_pred_) was used [8]. The predictive method utilizes logistic regression and is described by Terluin et al. [8]. The logistic regression is based on the dichotomized anchor response (importantly improved or not importantly improved) as the dependent variable and the PROM change score as the independent variable. The original MIC_pred_ was recently updated with an adjusted formula MIC_predadjust_ to increase the precision of the formula. The MIC_pred_ was most precise if the proportion of improved patients was 0.5. With the adjusted formula, the precision remained high when the proportion of improved patients were between 0.3 and 0.7. Furthermore, the updated formula considers the reliability of transition ratings [24]. Since DASH allows for three missing items, the calculation described by Terluin et al. was not possible, and therefore, missing items were replaced with mean values of the total score, allowing for estimation of the reliability of transition ratings [24].

Other commonly used anchor-based methods to determine MIC are the mean change method (MIC_mean_) and receiver operating characteristics (ROC) method (MIC_ROC_). To ease comparison between the predictive and traditional methods the three traditional methods were included as well. With the mean change method, the MIC_mean_ is estimated by calculating the mean score change of the patient subgroup that answered “Minimally improved” to the anchor question [2]. With the ROC method, we determined the MIC_ROC_ value according to the Youden criterion, discriminating patients from being importantly improved or not defined by the change in PROM score with the least degree of misclassification. 95% CI for MIC_ROC_ was determined using bootstrap replications (*n* = 1,000) [25].

A sensitivity analysis was conducted to investigate whether missing data would affect the scores of WOOS and the corresponding MIC values.

## Results

**Fig. 1:**
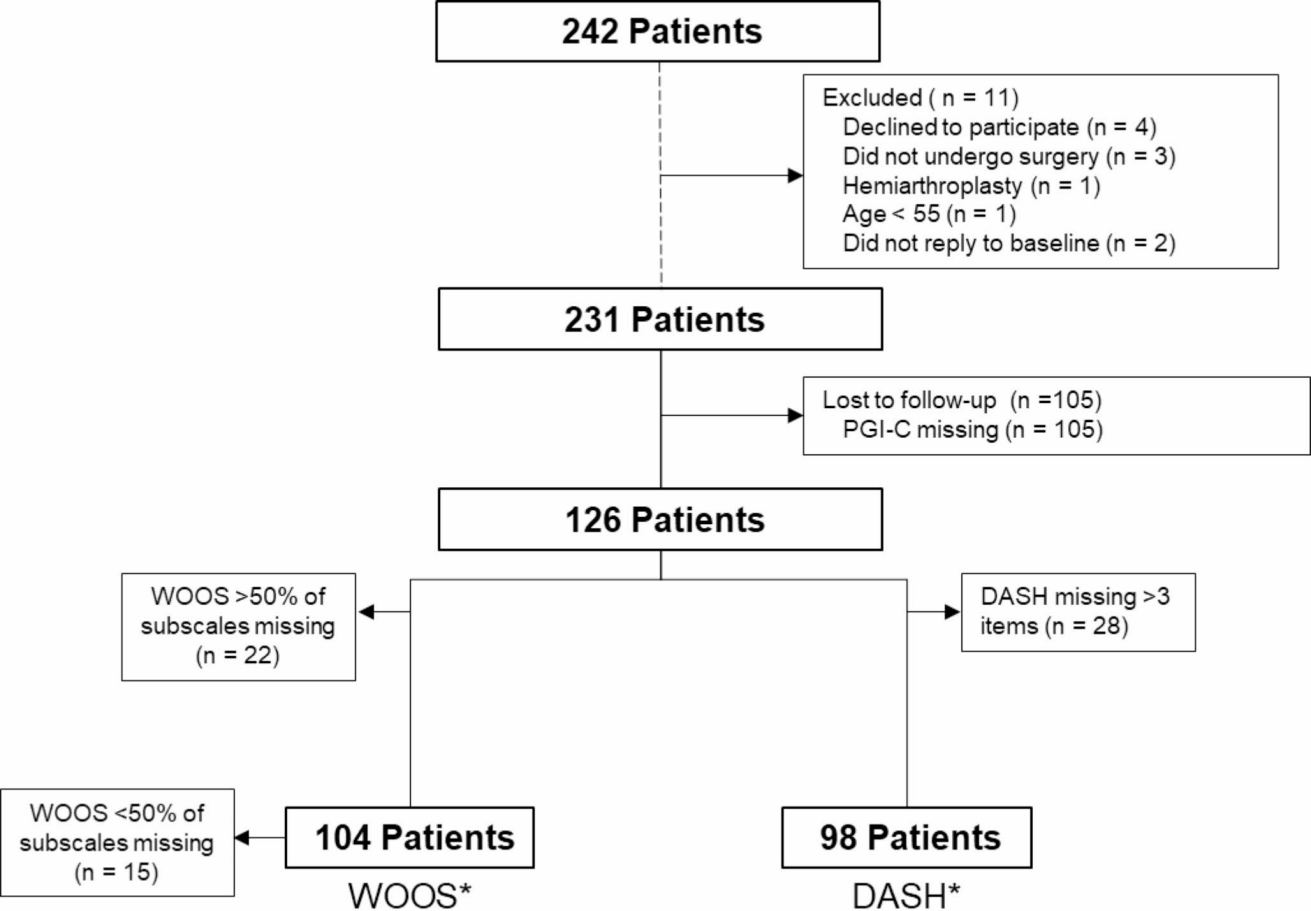
Flowchart of included patients. *Includes all patients that fulfilled the patient reported outcome measures preoperatively and at 3 months. WOOS: the Western Ontario Osteoarthritis of the Shoulder Index. DASH: disabilities of the arm, shoulder and hand questionnaire

Between March 2020 and January 2023, 231 patients underwent total shoulder arthroplasty, 105 patients had missing PGI-C, 22 patients had missing items in more than 50% of the subscales for WOOS and 28 had more than three items missing in DASH. This left 104 patients for the WOOS MIC analysis and 98 patients for the DASH MIC analysis (Fig. 1). No missing items in WOOS were present in 89 out of the 104 patients and were included in the sensitivity analysis. The patients had a mean age of 71 years, 56 had TSA and 48 had RSA (Table 2).

**Table 2:** Patient preoperative demographics

	Patients included in WOOS MIC analysis (*n* = 104)	Patients with missing PGI-C missing (*n* = 105)
Age, mean (SD)	71.0 (8.7)	69.1 (9.4)
Female, n (%)	58 (56)	60 (57.1)
Country DK, n (%)	59 (56.7)	19 (18.1)
Surgical type TSA, n (%)	56 (53.8)	38 (36.2)
Surgery in dominant arm, n (%)	66 (63.5)	63 (60.0)

### Descriptive data

The mean improvement in WOOS was 36.9 and 20.3 in DASH (Table 3). The overall proportion of patients reporting important improvements was 71%, while 27% reported either no change or minimal worsening, and 2% reported important worsening (Table 4). The mean improvement in WOOS in the importantly improved group was 45.6 and 15.5 in the not importantly improved group. For DASH, the mean improvement in the importantly improved group was 27.0 and 3.5 in the not importantly improved group (Table 5). The mean improvement for the surgical indication glenohumeral osteoarthritis for WOOS was 40.4 and 20.3 for DASH and for rotator cuff tear arthropathy the mean improvement for WOOS was 32.6 and 20.3 for DASH (Table 6).

**Table 3:** Preoperative, postoperative and change score for the patient reported outcome scores WOOS and DASH

	*n*	Mean	SD	Range
**WOOS**	104			
Preoperative		32	16	5–85
Postoperative		69	22	14–100
Difference		37	7	
**DASH**	98			
Preoperative		52	16	81 − 17
Postoperative		32	19	79 − 1
Difference		-20	3	

WOOS: the Western Ontario Osteoarthritis of the Shoulder Index. DASH: disabilities of the arm, shoulder and hand questionnaire

**Table 4:** Anchor based response classified into importantly improved or not importantly improved

	*n* (%)
**Importantly improved**	**74 (71)**
1. Very much improved	32 (31)
2. Much improved	42 (40)
**Not importantly improved**	**30 (29)**
3. Minimally improved	21 (20)
4. No Change	1 (1)
5. Minimally worse	6 (6)
6. Much Worse	2 (2)
7. Very much worse	0 (0)

**Table 5:** WOOS and DASH outcomes classified according to anchor-based groups

WOOS		Mean	SD	Range
Importantly improved (*n* = 74)	Preoperative	32	17	6–85
	Postoperative	78	17	23–100
	Difference	46	0.4	
Not importantly improved (*n* = 30)	Preoperative	30	12	5–51
	Postoperative	45	14	14–76
	Difference	16	3	
*DASH*				
Importantly improved (*n* = 70)	Preoperative	53	17	81–17
	Postoperative	26	17	73–1
	Difference	-27	-0.1	
Not importantly improved (*n* = 28)	Preoperative	50	15	78–21
	Postoperative	47	17	79–6
	Difference	-4	2	

WOOS: the Western Ontario Osteoarthritis of the Shoulder Index. DASH: disabilities of the arm, shoulder and hand questionnaire

**Table 6:** WOOS and DASH outcomes classified according to surgical indication

	Glenohumeral osteoarthritis (*n* = 56*)	Rotator cuff tear arthropathy (*n* = 46*)
**WOOS mean (SD)**		
Preoperative	30.9 (14.3)	32.4 (17.6)
Postoperative	71.3 (22.4)	65.1 (22.7)
Change	40.4 (8.1)	32.6 (4.1)
**DASH mean (SD)**		
**Preoperative**	49.7 (16.1)	55.2 (16.1)
Postoperative	29.3 (19.4)	34.9 (18.4)
Change	-20.3 (3.3)	-20.3 (2.3)

WOOS: the Western Ontario Osteoarthritis of the Shoulder Index. DASH: disabilities of the arm, shoulder and hand questionnaire. *Data was missing on indication and surgical type for 2 patients

### MIC values

The correlation between the change score of the WOOS and the anchor question was 0.6 and for the DASH 0.5. The correlations for WOOS and DASH were high enough to perform the MIC analysis. The reliability of transition ratings was 0.4 for WOOS and 0.6 for DASH. The MIC_predadjust_ values adjusted for the proportion of improved patients were 13.3 (-6.2; 23.3) for WOOS and 7.2 (12.8; 1.7) for DASH. The unadjusted MIC values determined with the predictive modelling approach MIC_pred,_ mean change method, and the ROC method varied across the used method with the adjusted MIC_predadjust_ value being lowest for WOOS and the MIC_mean_ being lowest for DASH (Table 7).

**Table 7:** Minimal important change (MIC) values determined with the predictive modeling approach unadjusted and adjusted, the mean change method and the ROC method. All estimates are presented as the estimated MIC values with a 95% confidence interval (CI)

	MIC_predadjust_ (CI)^a^	MIC_pred_ (CI)	MIC_mean_ (CI)	MIC_ROC_ (CI)
WOOS	13.3 (-6.2; 23.3)	30.2 (25.7; 34.3)	18.6 (11.8; 25.4)	26.7 (21.4; 43.0)
DASH	7.2 (12.8; 1.7)	15.3 (18.9; 11.6)	5.3 (12.5; -1.9)	14.2 (20.1; 4.0)

^a^Adjusted for the proportion of importantly improved and reliability of transition ratings. WOOS: the Western Ontario Osteoarthritis of the Shoulder Index. DASH: disabilities of the arm, shoulder and hand questionnaire

### Sensitivity analysis

The mean score did not change significantly when allowing for missing data in < 50% of each subscale. The MIC_predadjust_ did not differ either (Table 8).

**Table 8:** Complete case analysis on the WOOS score

	Complete WOOS (*n* = 89)	Total (*n* = 104)
**WOOS mean (SD)**		
Preoperative	31.9 (15.0)	31.6 (15.6)
Postoperative	69.7 (22.0)	68.5 (22.2)
Change	37.8 (7.0)	36.9 (6.6)
**MIC**_**predadjust**_ **(95% CI)**	16.1 (-5.8; 24.1)	13.3 (-6.2; 23.3)

WOOS: the Western Ontario Osteoarthritis of the Shoulder Index

## Discussion

To the best of our knowledge, this is the first study to use the predictive modeling approach to determine the MIC for the WOOS and DASH score in patients with treated with a total shoulder arthroplasty. We found a MIC_predadjust_ of 13.3 (95% CI -6.2; 23.3) for WOOS and 7.2 (95% CI 12.8; 1.7) for DASH, reflecting the smallest improvement needed to be considered important for the average patient three months after TSA or RSA. Allowing a few missing items in the WOOS score did not change the results of our analysis. The MIC estimates varied depending on the method used, the MIC for WOOS varied from 13.3 to 30.2 and DASH varied from 5.3 to 15.3.

Our adjusted MIC corresponds well to the previous studies that have determined MIC values for the WOOS. One study found a MIC for WOOS to be 12.3 using an anchor-based mean change method in patients with glenohumeral osteoarthritis treated with TSA at 1 year [10]. Two studies used the distribution-based methods and found a MIC between 8.2 and 14.2 with a follow-up varying from 1 to 10 years [9, 10]. The large variation in estimates can be due to methodological differences, and in particular, the distribution-based methods are not recommended to estimate the MIC as they do not account for the patient’s perspective [2].

One study used the predictive modeling approach to estimate MIC for DASH on patients with closed humeral shaft fracture treated both surgically and non-surgically and found a MIC of 9.4 (95% CI 10.5; 8.3) [13]. The results are on different upper extremity conditions but comparable to that found in our study (7.2 (95% CI 12.8; 1.7)). Other studies on different shoulder conditions have estimated a MIC for DASH to be between 4.4 and 12 [11–15]. Our results are within this range. With MIC estimates being highly dependent on the patient population, the intervention, and the time points, it is important to be transparent in the context and method used to estimate MIC.

MIC values can differ with both methodology and context [26]. The largest MIC value in this study was found using the predictive modelling approach and the smallest with the adjusted predictive modelling approach for WOOS and mean change method for DASH. The ROC values were higher than the mean change method and the adjusted predictive modelling approach. The ROC method is less precise and susceptible to errors, especially as the ROC method does not allow for adjustment, when the proportion of improved is not 50% [2, 8]. The adjusted predictive formula allows us to correct for the bias which can be introduced when proportion of improvement different from 50%. Furthermore, the formula was updated in 2022, allowing us to consider the reliability of transition ratings [2, 24]. The adjusted predictive and ROC methods should always be used over the mean change method when allowed, as the mean change method does not reflect a minimal threshold but rather a mean in a subgroup of patients [2].

It is important to remember that the MIC values reported in this study are considered to be context-specific and should be applied with care to other patient populations, time-points or interventions. The presented MIC values are considered applicable to other cohorts of patients with glenohumeral osteoarthritis and rotator cuff tear arthropathy undergoing TSA and RSA and comparable to demographics. MIC is the smallest improvement deemed relevant by patients and does not necessarily represent the best possible outcome or the full potential. Other determinants of the outcome after treatment may be relevant to evaluate in the comparison of different interventions. Further research is needed on the psychometric properties in relation to the utility and interpretability of WOOS and DASH in clinical trials.

### Limitations

There could be a risk of selection bias as 45% were lost to follow-up, did not have a PGI-C, or had too many missing items in WOOS and/or DASH. The reason for the high loss of follow-up was an error with the follow-up questionnaires at one of the sites, where patients did not receive the PGI-C, and thus were unable to answer it. The non-responders were similar to the responders in their baseline characteristics, and the differences were considered small with regards to age, preoperative and follow-up score on WOOS and DASH. Consequently, we do not expect that our MIC value would differ had we had a higher response rate. The mean age reported by the Danish Shoulder Arthroplasty Registry for patients with glenohumeral osteoarthritis and rotator cuff tear arthropathy is 70 and 75 years since 2004, which supports the representativeness of our sample [27]. There is a risk of recall bias when using an anchor question, as it may be difficult for the patient to recall previous severity levels. We chose a short follow-up period of three months, making it easier for patients to recall how their shoulder was prior to surgery. On the other hand, three months may not be a sufficiently long time period for all patients to recover fully after surgery, but our MIC was comparable to others with longer follow-up, suggesting the additional remission is small. With only 2% reporting to be much worse, the number of deteriorated patients was too low to establish a MIC value for the deterioration. Furthermore, our sample size did not allow separate MIC calculations for the TSA and RSA groups. The wide confidence intervals could be due to the sample size and the large heterogeneity within the patient group.

## Conclusion

The MIC_adjusted_ for improvement was 13.3 (95% CI -6.2; 23.3) for WOOS and 7.2 (95% CI 12.8; 1.7) for DASH at 3 months after total shoulder arthroplasty. The MIC values can be used to interpret longitudinal within-group score changes and determine the number of responders in studies or clinical trials.
